# Technology Adoption, Motivational Aspects, and Privacy Concerns of Wearables in the German Running Community: Field Study

**DOI:** 10.2196/mhealth.9623

**Published:** 2018-12-14

**Authors:** Martin Wiesner, Richard Zowalla, Julian Suleder, Maximilian Westers, Monika Pobiruchin

**Affiliations:** 1 Department of Medical Informatics Heilbronn University Heilbronn Germany; 2 Consumer Health Informatics Special Interest Group German Association for Medical Informatics, Biometry and Epidemiology Cologne Germany; 3 ERNW Research GmbH Heidelberg Germany; 4 GECKO Institute for Medicine, Informatics and Economics Heilbronn University Heilbronn Germany

**Keywords:** athlete, wearables, mobile phones, physical activity, activity monitoring

## Abstract

**Background:**

Despite the availability of a great variety of consumer-oriented wearable devices, perceived usefulness, user satisfaction, and privacy concerns have not been fully investigated in the field of wearable applications. It is not clear why healthy, active citizens equip themselves with wearable technology for running activities, and what privacy and data sharing features might influence their individual decisions.

**Objective:**

The primary aim of the study was to shed light on motivational and privacy aspects of wearable technology used by healthy, active citizens. A secondary aim was to reevaluate smart technology adoption within the running community in Germany in 2017 and to compare it with the results of other studies and our own study from 2016.

**Methods:**

A questionnaire was designed to assess what wearable technology is used by runners of different ages and sex. Data on motivational factors were also collected. The survey was conducted at a regional road race event in May 2017, paperless via a self-implemented app. The demographic parameters of the sample cohort were compared with the event’s official starter list. In addition, the validation included comparison with demographic parameters of the largest German running events in Berlin, Hamburg, and Frankfurt/Main. Binary logistic regression analysis was used to investigate whether age, sex, or course distance were associated with device use. The same method was applied to analyze whether a runner’s age was predictive of privacy concerns, openness to voluntary data sharing, and level of trust in one’s own body for runners not using wearables (ie, technological assistance considered unnecessary in this group).

**Results:**

A total of 845 questionnaires were collected. Use of technology for activity monitoring during events or training was prevalent (73.0%, 617/845) in this group. Male long-distance runners and runners in younger age groups (30-39 years: odds ratio [OR] 2.357, 95% CI 1.378-4.115; 40-49 years: OR 1.485, 95% CI 0.920-2.403) were more likely to use tracking devices, with ages 16 to 29 years as the reference group (OR 1). Where wearable technology was used, 42.0% (259/617) stated that they were not concerned if data might be shared by a device vendor without their consent. By contrast, 35.0% (216/617) of the participants would not accept this. In the case of voluntary sharing, runners preferred to exchange tracked data with friends (51.7%, 319/617), family members (43.4%, 268/617), or a physician (32.3%, 199/617). A large proportion (68.0%, 155/228) of runners not using technology stated that they preferred to trust what their own body was telling them rather than trust a device or an app (50-59 years: *P*<.001; 60-69 years: *P*=.008).

**Conclusions:**

A total of 136 distinct devices by 23 vendors or manufacturers and 17 running apps were identified. Out of 4, 3 runners (76.8%, 474/617) always trusted in the data tracked by their personal device. Data privacy concerns do, however, exist in the German running community, especially for older age groups (30-39 years: OR 1.041, 95% CI 0.371-0.905; 40-49 years: OR 1.421, 95% CI 0.813-2.506; 50-59 years: OR 2.076, 95% CI 1.813-3.686; 60-69 years: OR 2.394, 95% CI 0.957-6.183).

## Introduction

### Overview

Running has become one of the most popular exercise activities in western countries [1]. Technologically inclined runners find a great variety of wearable devices for the purpose of activity monitoring [2-5]. Research on device or app adoption and reasons for their use by runners seems to be underexplored in the literature, as reported by Evenson et al [6]. Motivation to either use or not use a wearable device for activity tracking is important, for example, for device vendors or health insurance companies to adjust their product strategies or incentive programs. For example, personal motivational factors can include “(a) Seeing if I met my goal (movement/sleep/calories count); (b) Look and feel good, improve mood and avoid sitting; and (c) Getting tips and recommendations” [7]. Other factors such as “technical failure or other technical problems, including empty batteries” can lead to negative experiences resulting in nonuse of wearables [8], and other reasons besides technology failure could also be a reason to abandon a particular device [9]. Despite the importance of understanding these factors, perceived usefulness, user satisfaction, and privacy concerns are under-investigated in the emerging field of consumer-centric mobile health (mHealth) applications. In this context, wearable technology such as Global Positioning System (GPS)–enabled sports watches and activity trackers is identified as a key trend in 2017 according to the worldwide survey of fitness trends [10,11]. However, little is known with respect to individual perceptions of the implications of activity data collection and possible sharing for a broad population of these technology users.

### Related Work

A number of studies in a variety of different settings have been performed. These include several studies that have investigated the accuracy of commercially available wearable devices, mostly in laboratory settings, for example, treadmill experiments [12-14]. These studies mainly focused on technical features and capabilities of the devices and results from small sample sizes and homogenous cohorts, that is, younger and active males have been reported.

A study conducted by Kaewkannate and Kim compared “the accuracy of four wearable devices in conjunction with user friendliness and satisfaction” [15], using a small cohort size (n=7), including 6 healthy male participants, and all participants being graduate students.

By contrast, Mercer et al focused on older adults living with chronic illnesses [16]. They applied a mixed-methods approach to study the usability and usefulness of wearable activity trackers. The authors found “wearable activity trackers are perceived as useful and acceptable” for adults aged over 50 years. A different study examined the “Feasibility of Fitness Tracking with Urban Youth” with a body mass index of 23 or higher [17]. The findings indicate that “wearable devices alone are not sufficient to support significant changes in existing physical activity practices” for users (n=24) in younger age groups. Nevertheless, feasibility studies indicate that “monitor comfort and design and feedback features [are] important factors to children and adolescents” [18].

Another study assessed the “acceptance and usage of wearable activity trackers in Canadian community-dwelling older adults” in a crossover design study [19]. For 20 adults, aged 55 years and older (mean 64 years), 2 wearable devices were given to participants who then rated different aspects of the devices and their use after 21 days of use. The authors report that “privacy was less of concern for older adults, but it may have stemmed from a lack of understanding of the privacy risks and implications.”

In other research, however, privacy seems to be an important aspect for users of wearable devices or apps [20]. Other researchers have found that “individuals' decisions to adopt healthcare wearable devices are determined by their risk-benefit analyses” [21]. The authors concluded that “individuals' perceived privacy risk is formed by health information sensitivity, personal innovativeness, legislative protection, and perceived prestige.” Their findings suggest that consumers’ motivations and buying decisions are “determined by [an individual] risk-benefit assessment.”

A review paper on ethical implications of user perceptions concluded that “wearable device users are highly concerned regarding privacy issues and consider informed consent as ‘very important’ when sharing information with third parties.” [22]. An explorative study including 82 participants investigated “privacy concerns and sensitivity regarding data gathered with wearables” [23]. The authors reported “that the participants would prefer to keep said data to themselves. Furthermore, user factors such as age, gender, and privacy behavior could not be identified as having an effect on sharing said data.” Yet, it remains an open question whether these findings are applicable to a broad and heterogeneous population, for example, a running community at a road running event.

Alley et al determined “people's current use, interest and preferences for advanced [pedometer] trackers” via a cross-sectional Australia-wide telephone survey [24]. The authors found that 31% of the participants “considered counting steps the most important function and 30% regarded accuracy as the most important characteristic.” About half of the participants were hesitant toward using current activity tracking devices or expressed individual skepticism. According to this survey [24], the main reasons “for not wanting to use a tracker were, ‘I don't think it would help me’ (39%), and ‘I don't want to increase my activity’ (47%).” It is not clear whether these findings can be confirmed in similar study settings in other countries.

### Aims of the Study

This study investigated several aspects of how citizens use smart technology for exercise activities. It was a follow-up study of previous research [4]. The primary study aim of the 2017 field study was to examine (a) reasons for use of wearable technology and (b) privacy concerns associated with the use of wearable technology. A secondary aim was (c) to study the current smart technology adoption within the running community in Germany and (d) to compare it with previous results from 2016.

This field study contributes to the mHealth field as it presents findings that originate from a real-world assessment and not from a potentially biased laboratory setting. In this context, the study cohort comprises participants from a public “Sport for All” road running event, that is, primarily physically active and healthy citizens of both sexes and all adult age groups (>16 years).

## Methods

### Study Design

The cross-sectional study consisted of 2 parts: a pre- and a postrace survey. The prerace survey aimed to answer research questions (a) to (c). It was scheduled for the registration period, that is, the day before and during the morning hours of the race day while runners picked up their number bibs and timing chips at the event site. For the postrace survey, the plan was to acquire data in the finisher area of the running event. Interviewer staff had the task of asking runners for individual step counts or the tracked distance in kilometers. As runners were quite exhausted after the race, no questions on motivational aspects, concerns, or willingness to share data with others could be posed at that time. At no point in time were individual, participant-related data, that is, name or address, collected.

### Study Setting—Road Running Event

The study was conducted during the 17th Heilbronner Trollinger Marathon on 6th to 7th May, 2017. The Trollinger Marathon is an annual road running event located in southern Germany [25]. In 2017, according to the official starter list [26], 6397 runners lined up for 4 different running courses: (1) full marathon, that is, 42.195 km, (2) half-marathon, that is, 21.0975 km, (3) walking/Nordic walking course with a length of 14.4 km, and (4) a marathon relay of approximately 3×14 km.

The event is part of the German Road Races Society calendar. Full and half-marathon courses conform to Association of International Marathons and Road Races (AIMS) and International Association of Athletics Federation (IAAF) regulations as both event categories are precisely measured by an accredited AIMS/IAAF Grade A or B measurer.

The prerace survey took place on May 6 (11:45 am to 5:30 pm) and May 7 (7:00 am to 10:00 am). Study staff were divided into several shifts, and it was ensured that at least two interviewers were present during registration hours. Interviewers were instructed to select runners randomly. Only runners older than the minimum participation age (ie, 16 years) were included in the cohort. Participation in the study was voluntary, that is, registered runners were asked whether they wanted to participate in a survey on wearable technology.

Due to bad weather conditions and heavy rainfall on May 7, the authors decided to cancel the postrace survey in the finisher area of the *Heilbronn Frankenstadion*. The main reason was that runners left the stadium quickly after crossing the finish line to escape the weather conditions and thus chances of acquiring a reasonable amount of study data were low.

### Questionnaire and Survey App

Participants were very focused on the registration and picking up their individual number bib, especially in the morning hours directly before the race. Therefore, questionnaires were designed in a compact and brief format.

Informed by the experiences from 2016, 2 questionnaires were designed:

1. Prerace questionnaire (Q_1_) contained items on (1) tracking devices used, (2) demographic data, for example, age and sex, (3) the running course chosen, (4) reasons for device or nondevice usage (eg, trust in own body or technical barriers), (5) parameters checked, (6) validity of collected or displayed data, (7) concerns regarding data privacy, and (8) voluntary data sharing,
2. Postrace questionnaire (Q_2_) to determine the accuracy of the tracked distances.

The items in Q_1_:

- were derived from existing literature; for items (4) and (5), see [24]; for item (6), see [27],
- have been posed to runners in the previous edition in 2016; for items (1) to (3), see [4], or
- have been raised by some study participants themselves during the previous study.

The developed questionnaire was tested with several staff members of nonresearch departments in our institution. We thereby checked the understandability and if it was feasible to conduct interviews with it.

All questionnaire items in Q_2_ were the same as in the previous study in 2016 to allow a comparison of the results in both years. English translations of Q_1_ and Q_2_ can be found in the Multimedia Appendices 1 and 2. An original German version of both questionnaires can be found as Multimedia Appendices 3 and 4.

**Figure 1:**
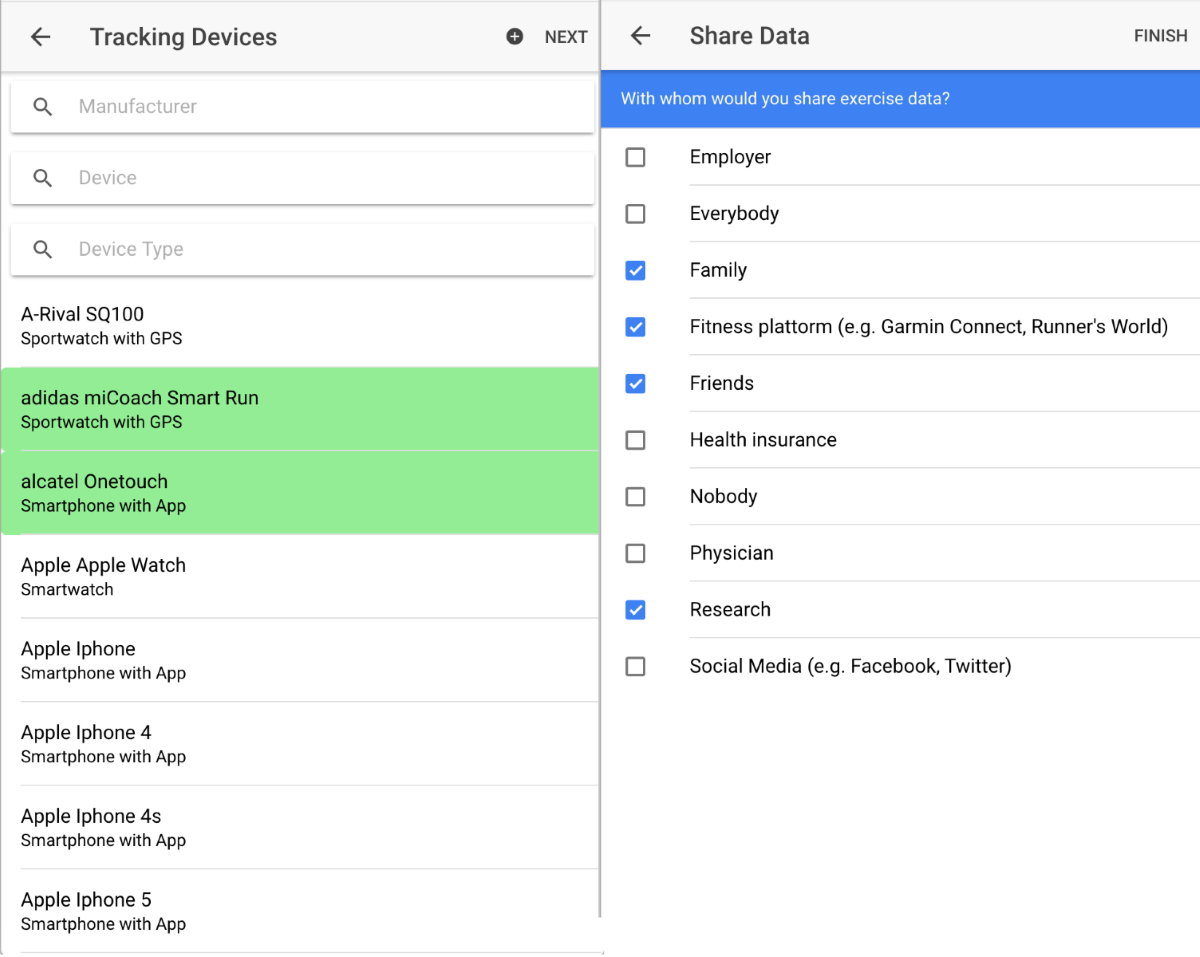
Selected screenshots of the Trolli survey app. Left: device selection from the list of all available devices and companion apps; right: preferences on sharing exercise data.

A relational database schema (see Multimedia Appendix 5) was derived from each questionnaire. The survey database— collected in 2016—on wearable devices and apps (156 distinct devices, 25 running apps, and 36 different vendors) was updated before the actual running event, and comprised 199 devices, 35 apps, and 37 vendors ahead of completion of the 2017 survey. A related Trolli survey app was implemented with the Ionic framework (in version 2.2.2) [28], depicted in Figure 1.

The app was deployed to the mobile phones (iOS: n=5, Android: n=10) of the interviewer staff and to 2 extra Android tablets. The completed questionnaires were stored on the internal disk of the mobile device and then synchronized via a *RESTful* Web service connected to the study database. If interviewers identified a previously unknown device, the creation of an entry for a new device was possible. Thereby, other interviewers could make use of it during subsequent interviews.

Both questionnaires (Q_1_ and Q_2_) were implemented electronically within this survey app. Neither personal data nor contact details of survey participants were collected. Therefore, the resulting records can be considered as an anonymized dataset that does not conflict with European, national, or federal data privacy laws. In case of technical issues such as power loss, loss of network connectivity, etc, paper-based backup copies of Q_1_ and Q_2_ were available on-site.

In 2016, during the transcription of the paper-based format, several questionnaires had to be excluded from the evaluation. In such cases, either (1) interviewers forgot to complete the questionnaire, (2) the handwriting of an interviewer was illegible, or (3) questionnaires provided nonspecific vendor or device information, for example, “a sports watch I bought at a supermarket”, and thus had to be excluded, which corresponded to a dropout rate of 7.79% (98/1258).

By contrast, in 2017, with support of a survey app, no incomplete questionnaires were encountered. As a consequence, no data had to be excluded from the later analysis. This corresponds to a dropout rate of 0.0% (0/845). Moreover, 28 new devices were captured on-site during the interviews.

### Statistical Analysis

The representativeness of the study cohort was analyzed via a comparison of age distributions of the full-marathon, half-marathon, and walking/Nordic walking against the official starter list—as provided by the event organizer in Heilbronn and the events in the German cities: Berlin, Frankfurt/Main, and Hamburg. No details on sex were available for relay runners in the Heilbronn starter list. Therefore, this group (n=41) was excluded from the analysis of representativeness. Chi-square analysis was applied to test whether the given age distribution of the Heilbronn starter list matched that of the study cohort (H_0_: distributions are equal, H_1_: distributions differ). If *P* ≥.05, H_0_ was accepted.

Important aspects for technology acceptance are trust in data and the protection of privacy [6,29,30]. Hence, these factors are of particular interest in the context of this study. Binary logistic regression was applied to analyze whether a runner’s age is a predictor for 3 factors: (1) trust in one’s own body, (2) privacy concerns, and (3) openness to sharing data. The same method was used to examine whether sex, age, or running course are predictors for wearable device usage.

Data were analyzed with the statistics software *R* [31] in version 3.3.3 (2017-03-06) on a Windows 10 Enterprise LTSB 2016/ 64-bit computer.

## Results

### Principal Findings

A total of 845 questionnaires were collected via our survey app and stored in the database. After verification of on-site data entries of previously unidentified manufacturers or devices (n=28), all 845 entries were included for further analyses. A comprehensive list of devices and manufacturers can be found in Multimedia Appendix 6.

### Study Cohort

The official starter list of the marathon (full and half) and walking/Nordic walking course comprised 6327 men and women. Male runners dominated the starter field for both full- and half-marathon, especially in the full-marathon starter field (82.6%, 514/622). However, more female runners (73.1%, 742/1015) were registered for the walking/Nordic walking course, as listed in Table 1.

Our study cohort covered 13.2% (845/6397) of the registered runners. Likewise, the sample covered 13.0% (611/4689) of the registered half-marathon runners. Chi-square analysis revealed that age distributions are not similar for this subcohort (*P*<.001). Nearly a quarter of all marathon runners were interviewed (23.5%, 146/622), which was representative for this subgroup (*P*=.55). However, our study cohort underrepresented walkers (*P*<.001), for which only 4.6% (47/1015) were included.

The age distribution of the Heilbronner Trollinger Marathon resembles those of larger running events, for example, the Berlin marathon (n=33,248 finishers in 2017 [32]), Berlin half-marathon (n=23,957 finishers in 2016 [33]), Hamburg marathon (n=11,930 finishers in 2017 [34]), Hamburg half-marathon (n=8299 finishers in 2017 [35]), Frankfurt/Main marathon (n=11,121 finishers in 2017 [36]), and Frankfurt/Main half-marathon (n=4558 finishers in 2018 [37]). Table 2 compares the age distributions of the aforementioned events with the starter list in Heilbronn 2017 on the basis of the respective finisher lists.

### Motivational Aspects

#### Usage and Nonusage

Runners who declared that they used one or more devices (73.0%, 617/845) for training or during running events were asked to select one or more reasons for doing so (see Multimedia Appendix 1). As presented in Table 3, nearly 9 out of 10 runners (89.8%, 554/617) used wearables as a tool for exercise control. For at least a third of the participants (34.0%, 210/617), technology was used for self-motivational reasons and as an enabler to *get more active* in general. Only a very small percentage (1.0%, 6/617) of the runners mentioned that they used a wearable device sponsored by their health insurance company/sickness fund or as recommended by their physician. The most common reason for technology use was to monitor exercise. This was consistent for runners across all age groups: 87.3% to 100% (see Table 3, Q_1_, No. 7; Multimedia Appendix 1).

**Figure 2:**
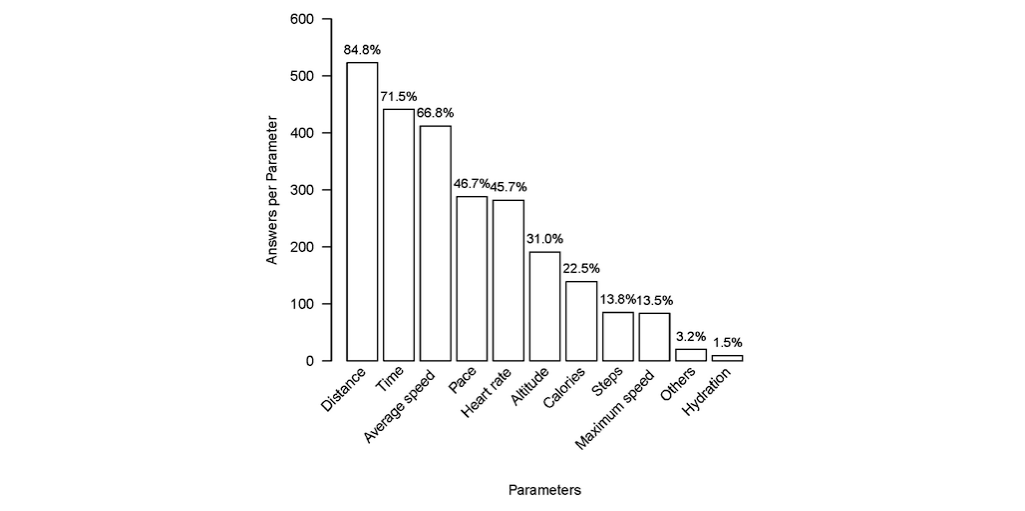
Answers given to Q1, No. 8 (n=2473) for each parameter (multiple answers possible). Numbers on the y-axis represent answers given per parameter; percentages at the top of each bar correspond to the relative proportion of runners (n=617) who selected one or more activity parameters.

If runners used a wearable device, several activity parameters were checked (see Figure 2). On average, 4 parameters were checked. The most frequent parameters were distance covered, time, and average speed. This corresponds to the most common reasons for using a device, that is, monitoring exercise (compare answers for No. 7 in Table 3). Monitoring of the hydration parameter seemed negligible.

Nontechnology users were asked why they did not use wearable devices. More than two-thirds (68.0%, 155/228) answered that they listen to their own body’ instead of technology. Technical barriers with wearables were reported by 12.7% (29/228); and 3.0% (7/228) had encountered bad experiences in the past.

As presented in Table 4, older age of nontechnology runners is associated with higher trust in one’s own body as the individual’s ‘measuring instrument’. This is a statistically significant finding for older age groups, that is, 50-59 years (*P*<.001) and 60-69 years (*P*=.008).

#### Validity and Data Sharing

##### Validity of Collected or Displayed Data

We asked runners if they trusted the data captured by their wearable device. Three out of 4 participants (76.8%, 474/617) stated that they always trusted the data. A fifth of participants (127/617) considered the visualized data as *partly* valid, whereas 1.8% (11/617) did not trust the data gathered at all.

**Table 1:** Distributions of sex and age groups (Pr_survey_) among runners for the full- and half-marathon and walking or Nordic walking. Pr_official_ denotes the proportion as given in the official starter list for the respective subcohort. Pr_official_ data were published by the event organizer of the Trollinger Marathon only as rounded percentage values, so precise n values for male and female age groups are unavailable. Furthermore, n=41 relay runners and runners with unknown course type were excluded.

Age groups per running course		Male (n)	Pr_survey_ (%)	Pr_official_ (%)	Female (n)	Pr_survey_ (%)	Pr_official_ (%)
**Marathon**		**N=121**			**N=25**		
	16-29	12	9.9	8.4	5	20	13.0
	30-39	17	14.1	20.2	7	28	17.6
	40-49	42	34.7	30.5	7	28	29.6
	50-59	37	30.6	29.6	4	16	31.5
	60-69	12	9.9	10.1	2	8	8.3
	70-79	1	0.8	1.2	0	0	0
	80+	0	0	0	0	0	0
	Unknown	0	0	0	0	0	0
**Half-marathon**		**N=400**			**N=211**		
	16-29	72	18.0	21.7	64	30.3	29.8
	30-39	89	22.3	27.5	43	20.4	28.1
	40-49	93	23.3	24.0	50	23.7	21.6
	50-59	113	28.3	20.4	41	19.4	16.8
	60-69	26	6.5	5.5	13	6.2	3.7
	70-79	6	1.5	0.8	0	0	0.1
	80+	1	0.3	0.03	0	0	0
	Unknown	0	0	0	0	0	0
**Walking or Nordic walking**		**N=12**			**N=35**		
	16-29	0	0	24.6	2	6	21.1
	30-39	0	0	25.0	3	9	23.3
	40-49	2	17	18.8	11	31	30.5
	50-59	5	42	16.9	11	31	16.2
	60-69	1	8	11.4	7	20	3.4
	70-79	4	33	3.3	1	3	0.5
	80+	0	0	0	0	0	0
	Unknown	0	0	0	0	0	0

**Table 2:** Age distributions of finishers at Berlin marathon 2017, Hamburg marathon 2017, Frankfurt/Main marathon 2017, Berlin half-marathon 2016, Hamburg half-marathon 2017, Frankfurt/Main half-marathon 2018 compared with registered runners in Heilbronn 2017.

Age groups per running course		Pr_Berlin_, n (%)	Pr_Hamburg_, n (%)	Pr_Frankfurt_, n (%)	Pr_Heilbronn_, n (%)
**Marathon**		**N=33,248**	**N=11,930**	**N=11,121**	**N=622**
	16-29	3950 (11.88)	1439 (12.06)	1425 (12.81)	57 (9.2)
	30-39	5221 (15.70)	3058 (25.63)	3128 (28.13)	123 (19.8)
	40-49	13,397 (40.29)	3886 (32.41)	3594 (32.32)	189 (30.4)
	50-59	8607 (25.89)	2880 (24.15)	2388 (21.47)	186 (29.9)
	60-69	1839 (5.53)	613 (5.14)	520 (4.68)	61 (9.8)
	70+	234 (0.70)	74 (0.62)	66 (0.59)	6 (1.0)
**Half-marathon**		**N=23,957**	**N=8299**	**N=4553**	**N=4689**
	16-29	4259 (17.78)	2241 (27.00)	795 (17.46)	1120 (23.89)
	30-39	6960 (29.05)	2726 (32.85)	1466 (32.20)	1299 (27.70)
	40-49	6561 (27.39)	1934 (23.30)	1231 (27.04)	1095 (23.35)
	50-59	4878 (20.36)	1138 (13.71)	873 (19.17)	909 (19.39)
	60-69	1124 (4.69)	225 (2.71)	167 (3.68)	236 (5.03)
	70+	175 (0.73)	35 (0.42)	20 (0.44)	30 (0.64)

**Table 3:** Answers given for selected questions (prerace questionnaire, Q_1_) on (non) motivation (No. 7, No. 6), privacy (No. 10), and data sharing (No. 11) by age group. Values in round brackets represent the proportion of runners who answered this question in the respective age group.

Answer options		Age groups (years), n (%)						Total
		16-29	30-39	40-49	50-59	60-69	70+	
**Users with device Q_1_, No. 7^a^, n=617^b^**		**N=192**	**N=245**	**N=268**	**N=262**	**N=47**	**N=9**	**N=1023**
	Gift	5 (4.2)	7 (4.8)	9 (5.4)	11 (7.4)	1 (3.1)	0 (0.0)	33
	Incentive program by health insurance	0 (0.0)	0 (0.0)	2 (1.2)	2 (1.3)	1 (3.1)	0 (0.0)	5
	Recommendation by physician/general practitioner	0 (0.0)	0 (0.0)	0 (0.0)	1 (0.7)	0 (0.0)	0 (0.0)	1
	Health aspects	12 (10.2)	13 (9.0)	20 (12.0)	33 (22.1)	5 (15.6)	1 (14.3)	84
	Self-motivation	50 (42.4)	57 (39.3)	54 (32.5)	44 (29.5)	5 (15.6)	0 (0.0)	210
	Curiosity	19 (16.1)	27 (18.6)	25 (15.1)	21 (14.1)	4 (12.5)	1 (14.3)	97
	Exercise control	103 (87.3)	131 (90.3)	151 (91.0)	132 (88.6)	30 (93.8)	7 (100.0)	554
	Trend setter	0 (0.0)	2 (1.4)	3 (1.8)	3 (2.0)	0 (0.0)	0 (0.0)	8
	Other	3 (2.5)	8 (5.5)	4 (2.4)	15 (10.1)	1 (3.1)	0 (0.0)	31
**Users without device Q_1_, No. 6^a^, n=228^b^**		**N=57**	**N=27**	**N=56**	**N=77**	**N=39**	**N=10**	**N=266**
	Costs	5 (10.2)	1 (4.0)	0 (0.0)	2 (2.9)	0 (0.0)	0 (0.0)	8
	Lack of trust	1 (2.0)	1 (4.0)	4 (8.7)	4 (5.9)	1 (3.0)	1 (14.3)	12
	Bad experiences	4 (8.2)	1 (4.0)	2 (4.3)	0 (0.0)	0 (0.0)	0 (0.0)	7
	Technical barriers	4 (8.2)	1 (4.0)	9 (19.6)	7 (10.3)	5 (15.2)	3 (42.9)	29
	I trust my body	24 (49.0)	17 (68.0)	30 (65.2)	55 (80.9)	26 (78.8)	3 (42.9)	155
	Other	13 (26.5)	3 (12.0)	10 (21.7)	7 (10.3)	5 (15.2)	3 (42.9)	41
	Don’t know	6 (12.2)	2 (8.0)	1 (2.2)	1 (1.5)	1 (3.0)	0 (0.0)	11
	Not stated	0 (0.0)	1 (4.0)	0 (0.0)	1 (1.5)	1 (3.0)	0 (0.0)	3
**Privacy concern Q_1_, No. 10, n=617^b^**		**N=118**	**N=145**	**N=166**	**N=149**	**N=32**	**N=7**	**N=617**
	Yes	31 (26.3)	42 (29.0)	59 (35.5)	68 (45.6)	14 (43.8)	2 (28.6)	216
	No	53 (44.9)	69 (47.6)	71 (42.8)	56 (37.6)	10 (31.3)	0 (0.0)	259
	Doesn’t matter	25 (21.2)	27 (18.6)	25 (15.1)	18 (12.1)	5 (15.6)	2 (28.6)	102
	Don’t know	9 (7.6)	7 (4.8)	11 (6.6)	7 (4.7)	3 (9.4)	3 (42.9)	40
**Data sharing Q_1_, No. 11^a^, n=617^b^**		**N=273**	**N=306**	**N=325**	**N=257**	**N=58**	**N=8**	**N=1227**
	Employer	3 (2.5)	3 (2.1)	2 (1.2)	1 (0.7)	0 (0.0)	0 (0.0)	9
	Physician	44 (37.3)	45 (31.0)	51 (30.7)	46 (30.9)	11 (34.4)	2 (28.6)	199
	Family	60 (50.8)	67 (46.2)	75 (45.2)	54 (36.2)	12 (37.5)	0 (0.0)	268
	Fitness platform	17 (14.4)	22 (15.2)	23 (13.9)	12 (8.1)	3 (9.4)	0 (0.0)	77
	Research	17 (14.4)	25 (17.2)	17 (10.2)	19 (12.8)	9 (28.1)	1 (14.3)	88
	Friends	78 (66.1)	83 (57.2)	88 (53.0)	58 (38.9)	9 (28.1)	3 (42.9)	319
	Health insurance	23 (19.5)	17 (11.7)	17 (10.2)	14 (9.4)	3 (9.4)	0 (0.0)	74
	Social media	5 (4.2)	10 (6.9)	7 (4.2)	5 (3.4)	2 (6.3)	0 (0.0)	29
	Everybody	14 (11.9)	10 (6.9)	11 (6.6)	6 (4.0)	0 (0.0)	0 (0.0)	41
	Nobody	12 (10.2)	24 (16.6)	34 (20.5)	42 (28.2)	9 (28.1)	2 (28.6)	123

^a^Denotes Questions in Q_1_ which allowed multiple answers.

^b^Number of runners, rather than number of responses, that is, can have multiple responses per runner.

**Table 4:** Binary logistic regression analysis of the parameter age for the factors: (1) Trust in own body (n=221, excluded: 7 participants in the age group 70 to 79 years), (2) Data sharing (n=617), and (3) Privacy concerns (n=474, excluded: “Doesn’t matter”, “Don’t know”, and 1 participant in the age group 70-79 years).

Model		Odds ratio (95% CI)	*P* value
**Trust in own body, age range (years)**			
	16-29 (Ref^a^)	1.0	—^b^
	30-39	2.214 (0.824-6.321)	.12
	40-49	1.953 (0.862-4.523)	.11
	50-59	4.407 (1.966-10.301)	<.001
	60-69	3.869 (1.470-11.203)	.09
	70-79	—	—
**Data sharing, age range (years)**			
	16-29 (Ref)	1.0	—
	30-39	0.570 (0.264-1.176)	.14
	40-49	0.440 (0.209-0.869)	.02
	50-59	0.288 (0.138-0.562)	<.001
	60-69	0.289 (0.109-0.783)	.01
	70-79	0.283 (0.054-2.123)	.16
**Privacy concerns, age range (years)**			
	16-29 (Ref)	1.0	—
	30-39	1.041 (0.371-0.905)	.89
	40-49	1.421 (0.813-2.506)	.22
	50-59	2.076 (1.183-3.686)	.01
	60-69	2.394 (0.957-6.183)	.06
	70-79	—	—

^a^Ref: Reference group in the respective regression model.

^b^Not applicable.

##### Privacy

Users of smart technology were asked about their views and opinions on nonvoluntary sharing of training or exercise data. In particular, this question included health insurance companies or device vendors, which could share such data for commercial or other purposes. As presented in Table 3, 2 out of 5 runners (42.0%, 259/617) stated that they would not be concerned if data were shared in such a manner. By contrast, 35.0% (216/617) said that they would not accept a vendor sharing data without their consent. Out of 617, 102 (16.5%) participants had a neutral perspective (“Doesn’t matter”) and only a small fraction of runners (6.5%, 40/617) were undecided (“Don’t know”). A detailed analysis revealed that runners in older age groups considered *privacy* a more important aspect of activity monitoring technology than younger users, see Table 4. This is a statistically significant finding for runners in the age group of 50 to 59 years (*P*=.01).

##### Data Sharing

In addition, we asked runners with whom they would share their personal training data on a voluntary basis. According to the results in Table 3, most participants that used technology preferred to share data only with their friends or family members (51.7%, 319/617 and 43.4%, 268/617). Nearly a third of the participants were open to sharing data with a physician (32.3%, 199/617). The public sharing of training data on social media platforms—for example, Twitter or Facebook—was only selected by 4.7% of the participants (29/617). One in every 7 runners (88/617, 14.3%) was open to sharing data for research purposes. Table 4 shows that voluntary data sharing with any other parties—that is, family, physician, employer, etc— decreases with higher age groups: 40 to 49 years (*P*=.02), 50 to 59 years (*P*<.001), and 60-69 years (*P*=.01).

### Device Categories

Devices used by runners were classified into 6 device categories: (D_1_) smartphones with related app, (D_2_) GPS-enabled sports watches, (D_3_) sports watches without GPS support, only heart rate monitors, (D_4_) smart watches, (D_5_) wristband activity trackers, and (D_6_) other devices. These are the same categories as in the 2016 study; hence, comparison with previous results can be made.

The results presented in Table 5 reveal that 228 out of 845 (27.0%) runners did not use a device. This represents a slightly larger proportion when compared with the results from 2016.

The most popular device was the Polar M400 (7.5%, 66/881). The GPS-enabled sports watch segment (D_2_) was dominated by the vendors Garmin (49.0%, 192/392) and Polar (31.9%, 125/392). If runners used their smartphone with a companion app, most of them preferred a model sold by Apple (49.4%, 78/158). The most popular running app was Runtastic/Runtastic Pro (63.9%, 101/158) followed by Nike+ Run Club, Strava, Sports Tracker and several other apps. These findings were very similar to the results from 2016 (see Table 2 in [4]). Devices in the categories D_4_ and D_5_ were found more frequently than in the previous year. However, these categories accounted for a smaller share than other device categories.

### Adoption of Wearable Devices

Regression analysis showed that use of wearable devices was associated with runners of younger age groups, see Table 6. This finding was statistically significant for 30 to 39 years age group (*P*=.002). Older age groups were less likely to use such devices. However, this finding was only significant for the age group of 60 to 69 years (*P*=.005). Being a participant of the walking/Nordic walking course was predictive of using no technology when compared with the reference group of half-marathon runners (*P*=.005). Marathon and relay runners were more likely to use wearable devices (odds ratio [OR] 1.368 and OR 1.458). Moreover, female runners seemed not to rely on technology (OR 0.745), although these findings were not statistically significant.

**Table 5:** Devices (D) used by category in 2017 compared with 2016 (n=653 devices used by 845 runners). Values in brackets denote the relative proportion of each category. Note: some runners (4.2%, 36/845) used more than one device.

Category	2017 (N=881), n (%)	2016 (N=978), n (%)
D_1_–Smartphone and app	158 (24.2)	181 (24.4)
D_2_–GPS^a^-equipped sports watch	392 (60.0)	437 (58.8)
D_3_–Heart rate monitor	25 (3.8)	37 (5.0)
D_4_–Smart watch	22 (3.4)	14 (1.9)
D_5_–Wristband activity tracker	33 (5.1)	28 (3.6)
D_6_–Other devices	23 (3.5)	47 (6.3)
No device	228 (27.0)	234 (26.1)

^a^GPS: Global Positioning System.

**Table 6:** Binary logistic regression of sex, age, and course type for the dependent variable “wearable device use” (n=845).

Feature		Odds ratio (95% CI)	*P* value
**Sex**			
	Male (Ref^a^)	1.0	—^b^
	Female	0.745 (0.528-1.054)	.09
**Age (years)**			
	16-29 (Ref)	1.0	—
	30-39	2.357 (1.378-4.115)	.002
	40-49	1.485 (0.920-2.403)	.11
	50-59	0.904 (0.572-1.424)	.67
	60-69	0.417 (0.226-0.765)	.01
	70-79	0.637 (0.188-2.243)	.47
	80+	<0.001	.98
**Course type**			
	Half-marathon (Ref)	1.0	—
	Marathon	1.368 (0.875-2.191)	.18
	Marathon relay	1.458 (0.875-2.191)	.51
	Walking or Nordic walking	0.391 (0.202-0.751)	.01
	Unknown	0.725 (0.280-2.032)	.52

^a^Ref: Reference group in the regression model.

^b^Not applicable.

## Discussion

### Principal Findings

One aim of the study was to gain insights into the reasons for use and privacy concerns of healthy active citizens with regard to wearable devices. The literature [19,21,23] yields an unclear picture whether privacy is a concern for using tracking technology. The results in this paper confirmed the plurality of opinions on data privacy and voluntary sharing aspects present in a heterogeneous population. Approximately 35% of runners raised concerns regarding whether vendors would share or sell their individual tracking data to third parties without explicit consent. By contrast, approximately 42% were not concerned and almost 17% did not care at all. Runners in older age groups considered privacy to be more important (50-59 years: *P*=.01) than in younger age groups. This is in line with our findings on voluntary data sharing (see Table 3), that is, openness to sharing activity data with family members, friends, and physicians, decreased for older age groups.

Our findings revealed that the primary reason for technology use is to monitor exercise levels (approximately 89.8%), followed by self-motivation (34.0%), curiosity (15.7%), and personal health aspects (13.6%). The main reason for using no technology at all was that runners prefer to “listen to their own body” (68.0%). The analysis showed that there are significant differences between age groups: when compared with runners in the 16 to 29 years age group, runners in the 50 to 59 years age group (*P*<.001) and 60 to 69 years age group (*P*=.008) had higher trust in listening to their own body feedback.

With respect to the second aim of the study, the analysis of adoption rates of wearables showed that 3 out of 4 runners used tracking technology. This is in line with our findings from the previous edition of the Trollinger Marathon study (see [4]). Most runners preferred to use a GPS-enabled sports watch (D_2_: 60.0%), followed by mobile phones with apps (D_1_: 24.2%). Smart watches (D_4_: 3.4%) and wristband activity trackers (D_5_: 5.1%) were less frequently used even though their relative share increased slightly compared with 2016. Overall, 76.8% of these runners stated that they always trust the tracking data of their personal device.

### Limitations

The data were collected through a cross-sectional survey, which may be subject to bias. For this reason, several limitations apply.

The cohorts for marathon and half-marathon runners were samples of randomly chosen registrants of the Trollinger Marathon. The age distribution of the event in Heilbronn was similar to those in Berlin, Hamburg and Frankfurt/Main. Age and sex distributions of the study sample were similar to the proportions published in the official starter list. However, Chi-square analysis revealed that only the marathon subcohort can be considered as representative for the respective group. Although statistical tests indicated no representativeness for the other subcohorts, we consider our data to be a valid sample, at least for the running community in (southwestern) Germany.

As in 2016, the response rate for the (Nordic) walking event was quite low (n=47, with a total of 1015 registered participants). The major part of the walkers were employees of the main sponsor of the event, and the handing out of number bibs for those participants was only conducted on May 7 and at a different location. More interviewer staff would have been necessary to cover this separate location, which was not feasible.

Questionnaires used in this survey were developed by the authors. Items in Q_1_ were either used in [4] or have been raised during interviews by study participants of the previous year (2016) or were derived from existing literature [24,27]. Therefore, we assume that Q_1_ achieves at least a moderate level of content-related validity. However, no evaluations on construct- or criterion-related validity and/or reliability were conducted, which poses a limitation for this study.

Due to heavy rainfall in the Heilbronn region on May 7, no postrace survey (Q_2_) on wearables’ accuracy could be conducted by our interviewer staff. Therefore, no results on the tracked course distances can be reported in the 2017 edition of the Trollinger Marathon study.

### Potential Pitfalls

In 2017, the deployment and use of our Trolli survey app at the event site helped to prevent or reduce (1) capturing data manually, (2) questionnaire transcription errors, (3) incomplete questionnaires, and (4) increase the postrace data analysis efficiency. Moreover, less time was needed to train the interviewing staff, and the handover between interviewer shifts was more streamlined, as the app could be preinstalled and tested individually. However, the mobile phones of some interviewers were outdated, which meant extra effort was required to set them up for the interviews on-site.

During crowded times in the registration area, the on-site cellular network was not able to handle all connection attempts initiated by numerous runners and our interviewer team. For such a scenario, unsent survey records were stored locally, and a built-in app feature allowed interviewers to resend those records to the study’s Web service. Unfortunately, for a small number of survey records, some interviewers initiated the transmission of a record multiple times, thereby skipping the server-side (asynchronous) response receipt. The resulting duplicates had to be identified and cleared afterward with the help of (1) server log files, (2) screenshots of related survey smartphones, and/or (3) time stamps available in the survey database. Checks for duplicates and stricter confirmation mechanisms were missing during the interview phase and could have prevented these issues.

### Comparison With Prior Work

The principal findings of the Trollinger Marathon of 2016 [4] on device adoption rates, usage of specific device categories, and the most popular devices and apps were replicated in 2017. In both editions, the results of binary logistic regression analysis support that younger age, male sex, and choice of long-distance running course are predictive of using technology in running activities.

Several studies on user acceptance of wearables exist [16,17,19,29,38-40]. However, these studies are based on surveys of between 16 and 260 participants that have a specific demographic background, for example, older adults or adolescents. By contrast, our study cohort included 845 users and nonusers of smart technology of both sexes and distributed over almost all age groups. The study revealed that runners without a device represent 27.0% (228/845) of all runners of which 12.7% (29/228) stated technical barriers as the primary reason for not using a wearable. When compared with the nonuser proportion caused by technological barriers (17.5%) reported in the study by Hermsen et al [8], the aforementioned fraction is slightly lower. The reason might be that the Trollinger study participants were given more answer options, in particular, “I trust my body”, “Lack of trust,” and the more general option “Bad experiences” (see Table 3, Q_1_, No. 6). Our results suggest that runners who do not use a device instead *use* their body to gather feedback (68.0%, 155/228).

A survey conducted by Deloitte among 2000 Germans in 2016 found that more than half of the participants (55%) were willing to share health data with a general practitioner [27]. By contrast, only 32.3% of the technology equipped runners of the sampled cohort were open to sharing exercise data with a physician. According to the Deloitte survey, a small fraction was open to sharing data with either device manufacturers (7%) or other internet companies (7%). This is comparable with our findings: fitness platforms (12.5%) or social media (4.7%) are channels to which users would upload their data.

Puri et al reported for 20 elderly people in Canada that “privacy was less of concern of older adults” and linked this to a potential “lack of understanding” [19]. This is not in line with our findings as runners of older age groups expressed privacy concerns more frequently than younger participants. We assume that runners who use wearable technology have at least a basic understanding on data collected and potential risks, supported by Huckvale et al [20]. However, a substantial fraction of the device users in our sample did not have concerns or reported that it did not matter (combined 58.5%) if their activity data were shared or sold by vendors to third parties. This particular finding slightly disagrees with the results of the study by Lidynia et al on privacy concerns. The authors report (n=82 German citizens—36 device users, 46 nonusers) “that the participants would prefer to keep said data to themselves.” [23]. According to their study, runners of older age groups are more hesitant to share data publicly, for example, via social media. This can be supported by our findings.

In a cross-sectional Australia-wide telephone survey (n=1257), Alley et al found that the “use of advanced trackers compared with pedometers was higher in males [...] and younger participants” [24]. The results of our logistic regression analysis on the same factors is in line with the findings from Australia.

The Online Eindhoven Running Survey 2014 (ERS14) comprised 2172 participants of the Half Marathon in Eindhoven [41]. In terms of adoption rates, the results were similar to our findings. In the ERS14 study, more than 86% of the participants used such a device, whereas 73% used at least one in the Trollinger study cohort. In the study by Janssen et al, the sports watch segment was also dominated by Garmin. However, a larger proportion of ERS14 participants used mobile phones in combination with apps (54.9%), which is not supported by our results (24.2%, see Table 5). This difference is likely to originate from the fact that the ERS14 study was conducted as an online survey and not as a field study at the site of an actual running event. Janssen et al reported age as a predictor for app and sports watch usage. Our results are in line with this finding: younger and middle-aged runners (16-29 years, 30-39 years, and 40-49 years) are more likely to use monitoring devices than runners in older age groups (50-59 years, 60-69 years, and 70-79 years).

Becker et al chose a qualitative approach with semistructured interviews (n=16) on factors influencing “continuous use of fitness trackers” [38]. They reported on perceived benefit, perceived privacy, perceived deficiency, and related subthemes. The interviews lasted 25 min on average. By contrast, for field studies—such as in our setting—and with a limited amount of time per interview, merely predefined answer options and short questionnaires were applicable. From a methodological perspective, it is important to keep in mind that runners are not willing to participate in long interviews on-site.

### Future Directions

Given that certain outcomes of the ERS14 study differ from those of our field study, it could be interesting for other researchers to replicate the design of this study in a different setting; that is, to analyze running communities in other countries. Moreover, a detailed study of female long-distance runners could provide new insights into their preferences toward tracking technology. Therefore, we encourage other international research groups to support the idea of interviewing runners at the site of an actual running event.

Alternatively, future surveys could investigate whether runners have changed their lifestyle, diet, or activity patterns as they started using a device for activity tracking. This could shed light on open questions, for example, “does wearable technology have long-term health effects besides being a stylish gadget?” or even “should health insurance companies promote wearable devices via incentive programs”?

Moreover, a question remains whether there is a link between *active* citizens and the openness toward collecting data and sharing it with others. Our study provides a first indication that both positions—openness and being concerned—exist within the German running community. These findings might, however, be different in other countries or communities.

In addition, it is still unclear whether citizens use wearable technology for other reasons than sport. This could, for instance, include monitoring of sleep, hydration, or blood glucose levels. In this context, other motivational patterns could be present and interesting for future studies.

### Conclusions

Use of technology for training or during running events is prevalent in the running community (approximately 73% in Southwestern Germany). Male long-distance runners and runners of younger age groups are more likely to use wearable devices. In total, 136 distinct devices by 23 vendors and 17 running apps were identified.

As expected, runners use wearable technology primarily for monitoring personal exercise levels (90%). The second most prevalent reason is self-motivation (34%), which is more important for younger runners. External incentives or recommendations are of marginal importance (<1%).

Three out of 4 runners always trusted the data tracked by their personal device. Two out of 5 runners (42%) explained that they were not concerned whether data collected by their device might be shared without explicit consent. By contrast, 35% said that they would not accept a vendor sharing data with third parties for commercial purposes. In the case of voluntary data sharing, runners preferred to give it to friends (52%), family members (43%), or a physician (32%), whereas only a small fraction (<2%) would give these data to their employer.

A large proportion (68%) of runners not using technology stated that they preferred to trust in the feedback from their own body. Approximately 11% answered that they experienced technical barriers when using wearables.

Future research might focus on preferences of female runners as they seem to be less likely (OR 0.745) to use tracking technology for running. Whether women perceive wearables and potential benefits differently from men remains a question open for research.

## Appendix

Multimedia Appendix 1 Prerace questionnaire Q1.

Multimedia Appendix 2 Postrace Questionnaire Q2.

Multimedia Appendix 3 Prerace questionnaire Q1 in the original German version.

Multimedia Appendix 4 Postrace questionnaire Q2 in the original German version.

Multimedia Appendix 5 Relational database schema of the Trolli 2017 study database.

Multimedia Appendix 6 Device categories, vendors, models, and apps.

## Abbreviations

AIMS: Association of International Marathons and Road Races
ERS14: Eindhoven Running Survey 2014
GPS: Global Positioning System
IAAF: International Association of Athletics Federation
mHealth: mobile health
OR: odds ratio
Q_1_: prerace questionnaire
Q_2_: postrace questionnaire
