# Supplementary material for: Technology Adoption, Motivational Aspects, and Privacy Concerns of Wearables in the German Running Community: Field Study
Source: JMIR Mhealth Uhealth. 2018 Dec 14;6(12):e201. doi: 10.2196/mhealth.9623 (PMC6315235; doi:10.2196/mhealth.9623)
Supplement: Multimedia Appendix 1 [file mhealth_v6i12e201_app1.pdf]

Multimedia Appendix 1: Pre-race Questionnaire Q1

Questions and response options of the pre-race questionnaire. Note: This is a translation of the original questionnaire in German language (see: Multimedia Appendix 5).

No.	***Question***	***Response options***
1	Do you use any devices for activity monitoring during running exercises or running running events?	Yes<br>No
2	*[IF Q1==yes]:*<br>Which kind of device do you use for exercising or during running events?	Selection from database of distinct devices and running apps<br><br>*Optional: Free text for vendor and device/app name if not available from the database.*
3	Sex	Male<br>Female<br>Not stated
4	Age	16-29<br>30-39<br>40-49<br>50-59<br>60-69<br>70-79<br>80+<br>Not stated
5	In which event do you participate in?	Walking<br>Marathon<br>Half-Marathon<br>Marathon Relay<br>Not stated
6	*[IF Q1 == no]:*<br>Why do you not use any device?<br><br>(multiple selection)	Costs<br>Lack of trust<br>Bad experiences<br>Technical barriers<br>Trust in own body<br>Other<br>Don’t know<br>Not stated
	[IF Q1 == yes]:<br>*Continue with Q7 to Q12*	
7	Why do you use wearable technology during running?<br>(multiple selection)	Gift<br>Incentive program by Health insurance<br>Recommendation by Physician / GP<br>Health aspects<br>Motivational aspects<br>Curiosity<br>Exercise control<br>Trend setter<br>Other
8	Which parameters do you check?<br><br>(multiple selection)	Distance<br>Average Speed<br>Average Pace (Time per Kilometer)<br>Fluid requirement<br>Heart rate

		Altitude difference<br>Calories<br>Maximum speed<br>Steps<br>Time<br>None<br>Other
9	Do you think the displayed values are reliable?	Always<br>Partly<br>No<br>Not stated
10	Do think it is a problematic issue that your exercise data could be transferred to third parties?	Yes<br>No<br>Doesn’t matter<br>Don’t know
11	With whom would you share exercise data?<br><br>(multiple selection)	Employer<br>Physician<br>Family<br>Fitness platform (e.g., Garmin Connect, Runners’ World)<br>Research<br>Friends<br>Health insurance<br>Social Media (e.g., Facebook, Twitter)<br>Everybody<br>Nobody
12	How many other devices for activity tracking do you own?	Number
