# Supplementary material for: Technology Adoption, Motivational Aspects, and Privacy Concerns of Wearables in the German Running Community: Field Study
Source: JMIR Mhealth Uhealth. 2018 Dec 14;6(12):e201. doi: 10.2196/mhealth.9623 (PMC6315235; doi:10.2196/mhealth.9623)
Supplement: Multimedia Appendix 2 [file mhealth_v6i12e201_app2.pdf]

Multimedia Appendix 2: Post-race Questionnaire Q2

Questions and response options of the post-race questionnaire. Note: This is a translation of the original questionnaire in German language (see Multimedia Appendix 6).

No.	***Question***	***Response options***
1	Which device did you use during the running event?	Selection from database of distinct devices and running apps<br><br>*Optional: Free text for vendor and device/app name if not available from the database.*
2	How did the device record the distance?	Kilometers<br>Steps
3	What distance was tracked by your device?	Kilometer count (2 decimals)<br>Step count
4	Sex	Male<br>Female
5	In which event did you participate in?	Half-Marathon<br>Marathon<br>Walking
