# Supplementary material for: Technology Adoption, Motivational Aspects, and Privacy Concerns of Wearables in the German Running Community: Field Study
Source: JMIR Mhealth Uhealth. 2018 Dec 14;6(12):e201. doi: 10.2196/mhealth.9623 (PMC6315235; doi:10.2196/mhealth.9623)
Supplement: Multimedia Appendix 3 [file mhealth_v6i12e201_app3.pdf]

Multimedia Appendix 3: Pre-race Questionnaire Q1 – Original German Version

Questions and response options of the pre-race questionnaire in German language.

Nr.	***Frage***	***Antwortmöglichkeiten***
1	Nutzen Sie beim Training oder während Laufevents Geräte zur Unterstützung…	Ja<br>Nein
2	*[WENN Q1==Ja]*:<br>Welche/ Welches Gerät(e) genau? (Hersteller, Modell gegebenenfalls App)	Auswahl aus Geräte- und Running-App Datenbank<br><br>*Optional: Freitext für Hersteller und Name des Gerätes bzw. der App, sofern nicht in Datenbank vorhanden.*
3	Geschlecht	Männlich<br>Weiblich<br>Keine Angabe
4	Alter	16-29<br>30-39<br>40-49<br>50-59<br>60-69<br>70-79<br>80+<br>Keine Angabe
5	An welchem Wettbewerb nehmen Sie teil?	Walking<br>Marathon<br>Halb-Marathon<br>Marathon-Staffel<br>Keine Angabe
6	*[WENN Q1 == Nein]:*<br>Warum nutzen Sie kein Gerät?<br><br>(Mehrfachnennungen sind möglich)	Kosten<br>Misstrauen<br>Schlechte Erfahrungen<br>Technische Hürden<br>Vertraue auf meinen Körper<br>Sonstige<br>Weiß nicht<br>Keine Angabe
	[WENN Q1 == Ja]:<br>*Weiter mit Q7 bis Q12*	
7	Warum nutzen Sie beim Laufen Technologie zur Unterstützung?<br>(Mehrfachnennungen sind möglich)	Anreiz/Geschenk<br>Bonusprogramm (Krankenkasse/Versicherung)<br>Empfehlung des Arztes<br>Gesundheitsaspekte<br>Motivation<br>Neugier<br>Trainingskontrolle<br>Trendsetter<br>Sonstige
8	Welchen Daten kontrollieren Sie?<br><br>(Mehrfachnennungen sind möglich)	Distanz<br>Durchschnittliche Geschwindigkeit<br>Durchschnittliche Pace (Zeit / Kilometer)<br>Flüssigkeitsbedarf<br>Herzfrequenz/Puls<br>Höhenmeter<br>Kalorien

		Maximale Geschwindigkeit<br>Schritte<br>Zeit<br>Keine<br>Sonstige
9	Halten Sie die angezeigten Werte für zuverlässig?	Immer<br>Teils teils<br>Nein<br>Keine Angabe
10	Halten Sie es für problematisch, dass Ihre gesammelten Trainingsdaten an Dritte weitergegeben werden können?	Ja<br>Nein<br>Ist mir egal<br>Weiß nicht
11	Mit wem würden Sie Trainingsdaten teilen?<br><br>(Mehrfachnennungen sind möglich)	Arbeitgeber<br>Arzt<br>Familie<br>Fitnessplattform (z.B. Garmin Connect, Runners' World)<br>Forschung<br>Freunde<br>Krankenkasse<br>Social Media (z.B. Facebook, Twitter)<br>Jedem<br>Niemandem
12	Wie viele weitere Geräte zum Erfassen von Fitness-aktivitäten besitzen Sie?	Anzahl
