# Supplementary material for: Technology Adoption, Motivational Aspects, and Privacy Concerns of Wearables in the German Running Community: Field Study
Source: JMIR Mhealth Uhealth. 2018 Dec 14;6(12):e201. doi: 10.2196/mhealth.9623 (PMC6315235; doi:10.2196/mhealth.9623)
Supplement: Multimedia Appendix 4 [file mhealth_v6i12e201_app4.pdf]

Multimedia Appendix 4: Post-race Questionnaire Q2 – Original German Version

Questions and response options of the post-race questionnaire in German language.

Nr.	***Frage***	***Antwortmöglichkeiten***
1	Welches Gerät haben Sie genutzt?	Auswahl aus Geräte- und Running-App Datenbank<br><br>*Optional: Freitext für Hersteller und Name des Gerätes bzw. der App, sofern nicht in Datenbank vorhanden.*
2	Wie hat das Gerät die Distanz gemessen?	Kilometer<br>Schritte
3	Welche Distanz wurde gemessen?	Anzahl Kilometer (2 Nachkommastellen)<br>Anzahl Schritte
4	Geschlecht	Männlich<br>Weiblich
5	An welchem Wettbewerb nahmen Sie teil?	Halb-Marathon<br>Marathon<br>Walking
