# Supplementary material for: Technology Adoption, Motivational Aspects, and Privacy Concerns of Wearables in the German Running Community: Field Study
Source: JMIR Mhealth Uhealth. 2018 Dec 14;6(12):e201. doi: 10.2196/mhealth.9623 (PMC6315235; doi:10.2196/mhealth.9623)
Supplement: Multimedia Appendix 6 [file mhealth_v6i12e201_app6.pdf]

Multimedia Appendix 6: Device Categories, Vendors, Models and Apps

Device categories, vendors, models and apps used by runners as found in the Trolli survey. Values in curved brackets represent the number of occurrences for the respective category, vendor, device or app.

Category	*Vendors*	*Devices*
**D_1 –** Mobile phone & App (158)	Apple (78), Samsung (46), Sony (10), HTC (7), Huawei (6), LG (3), Motorola (3), Nokia (3), Google (1), Wiko (1)	Iphone 6 (19), Iphone 7 (17), Iphone 6s (15), Galaxy S7 (9), Galaxy S5 (8), Iphone SE (7), Iphone 5s (7), Huawei - other (6), Iphone 5 (5), Galaxy A3 (5), Galaxy S4 mini (4), Galaxy Note 4 (3), Xperia Z3 (3), Iphone 4 (3), Sony - Other (3), Iphone (3), Nokia - other (3), Xperia (2), Iphone 4s (2), Galaxy S3 (2), Galaxy J5 (2), One (2), 10 (2), Samsung - other (2), Galaxy S6 (2), Galaxy S4 (2), Galaxy S6 Edge (2), Galaxy A5 (2), Galaxy S3 mini (2), Xperia L (1), Razr (1), K10 (1), Nexus 5X (1), HTC - other (1), Jerry (1), Xperia Z5 Compact (1), Desire (1), G4 (1), One X+ (1), LG - other (1), Moto G4 (1), Motorola - Other (1), Galaxy S7 edge (1)
		Apps
		Runtastic (76), Runtastic Pro (25), Nike+ Run Club (14), Other App (8), Runkeeper (7), Sports Tracker (7), Strava (7), Map My Run (3), S Health (3), Sportractive - Laufen Joggen (3), Polar beat (2), Apple Health (1), Endomondo (1), komoot (1), My Asics (1), Runmeter (1), Tourenapp Dt. Alpenverein (1)
Category	***Vendors***	***Devices***
**D_2 –** GPS sports watch (392**)**	Garmin (192), Polar (125), TomTom (46), Suunto (20), Fitbit (6), Crane (1), Other (1), Sigma (1)	M400 (66), Fenix 3 (23), V800 (21), Garmin - other (17), Forerunner 305 (14), Polar - other (13), Forerunner 310XT (13), Forerunner 920XT (13), Forerunner 235 WHR (12), vivoactive HR (10), Runner (10), TomTom - other (9), Forerunner (9), M200 (9), Forerunner 230 (9), Forerunner 205 (8), Runner Cardio (8), Ambit 2 S HR (8), Forerunner 220 (8), Runner 2 Cardio (7), Forerunner 610 (7), Runner 2 (7), RCX3 (7), Forerunner 910XT HR (6), vivoactive (6), Surge (6), Ambit 3 Peak (5), Forerunner 35 (5), RCX5 (4), Forerunner 210 HR (4), Suunto - other (4), Forerunner 620 (4), Forerunner 735XT (4), Forerunner 110 HR (4), Forerunner 225 (4), Forerunner 10 (3), Forerunner 405 (3), Multisport (3), RC3 (2), RS300X (2), Forerunner 410 HR (2), Ambit 3 Vertical (2), Fenix 5 (2), GPS-sport watch other (2), Forerunner 25 (1), Quest (1), Sigma - other (1), Runner 3 (1), Forerunner 630 HR (1), RS800CX (1), Nike+ SportWatch (1)
**D_3 –** Heart rate monitor (25)	Polar (19), Sigma (3), Other (2), Timex (1)	Polar heart rate monitor (6), A300 (2), RS100 (2), M91 ti (2), Heart rate monitor RC14.11 (2), Heart rate monitor /Cardio other (2), RS200sd (2), Ironman Race Trainer Pro (1), FT7 (1), Heart rate monitor Pc15.11

		(1), S720i (1), FT4 (1), FT40 (1), FT1 (1),
**D_4 –** Smart watch (22)	Apple (16), Samsung (4), Fitbit (2)	Apple Watch (16), Blaze (2), Gear S2 (2), Gear Fit 2 (2)
**D_5 –** Wristband Activity Tracker (33)	Garmin (13), Fitbit (8), Polar (8), Other (2), Mio (1), Epson (1)	Charge 2 (6), vivosmart HR (5), A360 (5), Loop (3), vivofit (3), vivosmart HR+ (2), Activity tracker – other (2), vivofit 2 (2), FUSE (1), PS-500 (1), Alta (1), Charge HR (1), Garmin activity tracker – other (1)
**D_6 –** Other Devices (23)	Other (15), Casio (2), Fitbit (2), Polar (2), Garmin (1), Decathlon (1)	Stopwatch (5), Watch (6), Unknown device (5), Watch (4), One Activity- and Sleep-Tracker (2), Approach G10 (1), mp3-Player (1), Decathlon - pedometer (1), Chest Strap (1), M450 (1)
